# Serratus anterior plane block for minimal invasive heart surgery

**DOI:** 10.1186/s12871-018-0614-5

**Published:** 2018-10-20

**Authors:** Vivien Berthoud, Omar Ellouze, Maxime Nguyen, Maria Konstantinou, Serge Aho, Ghislain Malapert, Claude Girard, Pierre-Gregoire Guinot, Olivier Bouchot, Belaid Bouhemad

**Affiliations:** 1 Unité d’Anesthesie Réanimation Cardio-Vasculaire, CHU François Mitterrand, BP 77908, 21709 Dijon Cedex, France; 2 Service d’Epidémiologie et d’Hygiène Hospitalières, CHU François Mitterrand, BP 77908, 21709 Dijon Cedex, France; 3 Service de Chirurgie Cardiaque, Vasculaire et Thoracique, CHU François Mitterrand, BP 77908, 21709 Dijon Cedex, France

**Keywords:** Serratus anterior plane block, Pain, Post operative, Minimal invasive heart surgery, Thoracotomy

## Abstract

**Background:**

Minimal invasive heart surgery (MIHS) presents several benefits, but provides intense and prolonged post-operative pain. Our objective was to compare efficacy of serratus anterior plane block (SAPB) with continuous wound infiltration (CWI) for management of post-operative pain following MIHS.

**Methods:**

It’s retrospective, monocentric study between November 2016 to April 2017. The study was performed at the University hospital of Dijon, Burgundy, France. All patients scheduled for MIHS was included. Data was collected retrospectively. During this period, 20 patients had SAPB and 26 had CWI. SAPB was performed before extubation with a single injection of 0.5 mg/kg of ropivacaïne (5 mg/ml). In the CWI group, catheter was inserted in the subcutaneous space by the surgeon at the end of the procedure. A 10 ml bolus of ropivacaïne (7.5 mg/mL) was followed by a continuous infusion (2 mg/ml) between 7 and 12 ml/h for 48 h. Morphine consumption and visual analog score (VAS) were recorded for 48 h. Length of stay in intensive care unit and hospital was also collected.

**Results:**

Morphine consumption and VAS score were significantly lower in SAPB group (*p* < 0.01). Length of stay in intensive care and hospital was significantly was decreased in SAPB group.

**Conclusion:**

SAPB appears effective in reducing postoperative MIHS pain.

**Electronic supplementary material:**

The online version of this article (10.1186/s12871-018-0614-5) contains supplementary material, which is available to authorized users.

## Background

Minimally-invasive heart surgery (MIHS) for aortic, mitral valve replacement or repair has developed rapidly. It presents several advantages including decreasing the risk of mediastinitis, leaving a more esthetic scar, facilitating post-operative rehabilitation and reducing hospital length of stay, compared with sternotomy [1]. MIHS requires thoracic incision in the right 4th or 5th intercostal space and is followed by intense and prolonged post-operative pain [2, 3]. The pain is exacerbated by breathing movements, coughing and respiratory physiotherapy. It is most severe during the first 48 h after surgery with repercussions on breathing, cognitive, urinary and digestive function, and may prevent early rehabilitation.

Usually, pain is treated with morphine agonist, which also have many side effects, affecting the duration of post-operative sedation, hemodynamic stability and respiratory function, particularly in elderly patients [4, 5].

The management of pain requires a multimodal approach that associates neuraxial anesthesia (spinal or thoracic epidural anesthesia), systemic opiates and step I or II analgesics [6, 7]. The use of the perimedullary technique for analgesia in cardiac surgery is limited by the fear of perimedullary hematoma [8, 9]. Continuous wound infiltration (CWI) analgesia is an effective practice for management of postoperative pain in a multimodal approach, especially during thoracotomy, but which is probably still insufficient [10].

Serratus anterior plane block (SAPB) is a recently described technique for chest wall analgesia. SAPB provide anesthesia of dermatomes T2 to T9, where surgical incision is realized (4th or 5th right intercostal space). The procedure consists of injecting local anesthetic between the muscle planes of the chest wall to block the lateral cutaneous branches of the intercostal nerves [11]. SAPB has already shown its efficacy in the management of post-operative pain following breast surgery, thoracoscopy, thoracotomy and multiple rib fractures [12–16]. No data has yet been published on the effectiveness of analgesia by SAPB for MIHS.

Several loco-regional analgesic techniques are available for postoperative analgesia of thoracotomy. Currently, in minimally invasive cardiac surgery no study has compared CWI c SAPB. Because the serratus block allows extensive analgesia, our hypothesis is that SAPB is superior to CWI in terms of analgesia. Our objective was to demonstrate the superiority of SAPB compared to CWI in the management of post-operative pain following MIHS.

## Methods

### Study design

We performed a retrospective analysis of the data of consecutive patients operated from November 2016 to April 2017 at our institution. Before implementing this new technique routinely and analyzing our data on these patients, we performed SABP with setting systematically a CWI and a morphine patient controlled analgesia in case of inefficiency of the SABP. Decision to realize SABP was done in agreement with the surgical team and some of the anesthesiologists who realize ultrasound guided regional anesthesia. Accordance to french law, an ethical committee is not necessary to realize a new locoregional analgesia technique, and this is part of the professional practices prerogatives. Nevertheless, we systematically inform all patients of the postoperative analgesia technique that will be performed, including this new technique (SABP) during preoperative anesthesia consultation, but no written consent was collected. Because, the study is a retrospective analysis of institutional data, and in compliance with the French law on clinical research, we only had to obtain French personal data protection authorization. We submitted our study to the national commission for data protection (CNIL) and obtained authorization 2,053,943 v 0. The present report was drafted in line with the Strengthening Reporting of Observational Studies in Epidemiology statement for cohort studies.

The inclusion criteria were patients who underwent MISH. Exclusion criteria were: patients under 18 years old, pregnancy, allergies to local anesthesics, cognitive dysfunction, and post-operative ventilation during ≥24 h.

### Anesthesia protocol

In our unit, anesthesic and post-operative management is protocolized. Anesthesia consultation was carried out at least 48 h before the intervention, and premedication consisted of oral gabapentin 300 mg the day before and the morning of the surgery. Anesthesia was given accordingly to protocol in use. After pre-oxygenation with 100% inspired oxygen, anesthesia was performed with propofol, sufentanil and cisatracurium for obtained bispectral index between 60 to 40 and neuromuscular transmission (NMT) = 0/4. Orotracheal intubation (OTI) was performed with a double-lumen endotracheal tube. Post-operative pain was managed by continuous CWI or SAPB. Group selection was based on the anesthetist in charge of the patient and familiar with SABP.

The CWI catheter was inserted into the subcutaneous space by the surgeon at the end of the intervention. The catheter was purged with a bolus of 10 ml of ropivacaïne at 7.5 mg/ml. An elastomeric pump containing 300 ml of ropivacaïne at 2 mg/ml was set up allowing a continuous infusion at 7 mL/h for 48 h. The flow rate was increased up to 12 ml/h if the pain NRS was > 3.

**Fig. 1:**
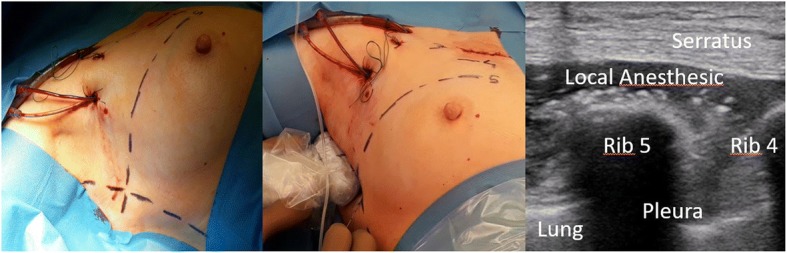
SAPB landmarks, punction, and echographic view

The SAPB was performed by the anesthetist (medical team) at the end of the surgery. Ultrasonography was used with the transducer placed against the midaxillary line to visualize the pleura, the lateral arch of the 4th or 5th rib and the different muscle planes from the most superficial to the deepest: the latissimus dorsi muscle, the serratus anterior muscle and the intercostal muscles. The needle (VYGON locoplex® 50 or 100 mm) was inserted in the plane of the transducer into the 4th or 5th intercostal space, the progress of the needle was followed on the screen until it reached the space between the serratus anterior muscle and the intercostal muscles. A single injection of 0.5 ml/kg of ropivacaïne 5 mg/ml was given without exceeding a maximal dose of 200 mg. During the injection, we looked for the formation of a bi-convex lens shape between the two muscles (Fig. 1).

At the end of surgery, all patients were sedated with propofol and the lungs were mechanically ventilated until haemodynamic stability and normothermia were obtained and blood loss was considered acceptable (less than 1 ml kg^− 1^ h^− 1^). After obtaining the 3 criteria above, extubation is carried out according to the protocol of the service. Every patient had an intravenous titration of morphine with a bolus of 2 to 3 mg every 7 min from extubation, until a pain visual analogue scale (NRS) below 3 was achieved. Then PCA morphine was set up with the following parameters: 1 mg bolus, refractory period of 7 min, maximum dose of 20 mg every 4 h and without continuous infusion. In addition, the patients were systematically given 1 g of intravenous paracetamol every 6 h on day 0 and then orally from day 1. The use of other analgesics if the pain NRS remained above 3 was left to the discretion of the physician.

The following data we retrieved from the data base: morphine consumption, at NRS h0, h6, h24, use of additional analgesics (ketoprofen, tramadol or nefopan), nausea and vomiting episode, need for non-invasive ventilation (NIV), during the first 48 h after surgery. We also recorded length of ICU and hospital stay. All study data are available in an additional file attached whit the manuscript (see Additional file 1).

### Statistical analysis

Two groups of patients were compared: patient with CWI and patient with SAPB.

Quantitative data were expressed as means and standard deviations for variables with normal distribution and as medians and interquartile ranges (IQR) otherwise. Qualitative data were expressed as numbers and frequencies. Quantitative data are shown in box plots and were compared using the appropriate statistical test (Student’s test or a non-parametric Mann-Whitney Wilcoxon test depending on the properties of the variable). Qualitative data were compared using the appropriate statistical test (Chi-2 with or without correction for continuity or Fisher’s exact test depending on the properties of the variable).

A value *p* ≤ 0.05 was considered significant for differences between the two groups. StataCorp 14.0 (Statacorp 4905 Lakeway Drive College Station, TX 77845 USA) was used for the statistical analysis.

## Results

### Baseline characteristics

During the observational period, 345 patients were screened in cardiac surgery, 48 patients have operated by MISH, and 2 patients were excluded for prolonged mechanical ventilation more than 24 h postoperatively, 1 for acute respiratory distress syndrome and one for hemorrhagic shock with surgical revision. We included 26 patients in CWI group and 20 SAPB.

The baseline characteristics are presented in Table 1. There were no significant baseline differences between the two groups.

**Table 1:** General characteristics of the studied groups

	CWI group (*n* = 26)	SAPB group (*n* = 20)	*p* Value
Sex ratio (M/F)	18/8	10/10	0.23
Age	67 [64–72]	67 [60–74]	0.78
BMI (kg/m^2^)	27 [25–29]	29 [23–31]	0.50
Euroscore 2	1.2 [0.8–2.0]	0.9 [0.7–1.6]	0.20
Respiratory disease	4 (15)	4 (20)	0.70
Characteristics of the surgery			
AVR	21 (81)	14 (70)	0.49
MVR	4 (15.4)	0	0.12
Mitral valve plasty	1 (3.9)	5 (25)	0.07
CABG	0	1 (5)	0.46
Duration of surgery (min)	192 [170–227]	194 [167–216]	0.62
Intraoperative data			
Gabapentin	25 (96)	19 (94)	0.76
Ketamine	8 (31)	7 (35)	0.50
Sufentanil (μg)	103 [90–118]	108 [94–128]	0.26
Duration anesthesia (h)	10 [9–11]	9 [9–10]	0.48
Time to extubation (h)	5 [4–6]	5 [4–6]	0.90

Date are presented as median [IQR] or n (%)

*AVR* aortic valve replacement, *MVR* mitral valve replacement, *CABG* coronary artery bypass graft surgery, *Euroscore 2* European System for Cardiac Operative Risk Evaluation: risk model which allows the calculation of the risk of death after a heart operation

### Primary outcome results

The perioperative dose of sufentanil was comparable in the two groups with a median of 103 μg [90–118] in the CWI group and 108 μg [94–128] in the SABP group (*p* = 0.26). The median consumption of morphine (mg) in the CWI and SAPB group was 21 versus 11 mg, respectively, *p* < 0.01 (Table 2).

**Table 2:** Morphine consumption, NRS and additionnal analgesics during the first 48 h in the two studied groups

	CWI group (*n* = 26)	SABP group (*n* = 20)	*p* Value
Dose of morphine (mg)			
Total to 48 h	21 [11–36]	11 [5–19]	< 0.01
Titration (h0)	5 [4–8]	2 [0–3.5]	< 0.01
h0-h6	10 [7–15]	7 [4–10]	0.01
h0-h24	18 [10–31]	10 [6–13]	< 0.01
NRS			
h0	5 [4–6]	0 [0–4]	< 0.01
h6	2 [0–4]	0 [0–3]	0.13
h24	0.5 [0–3]	0 [0–2]	0.41
h48	0 [0–2]	0 [0–2]	0.85
Additional analgesics			
Nefopam	6 (23)	1 (5)	0.20
Tramadol	5 (19)	0 (0)	0.06
Ketoprofen	4 (15)	5 (25)	0.66
Nausea and vomiting	8 (30)	5 (25)	0.7
NIV	6 (23)	3 (15)	0.7
Length of stay			
ICU (h)	41 [25–69]	24 [20–42]	0.03
Hospital (days)	8 [7–9]	6 [5–8]	0.03

Data are presented as median [IQR] or n (%)

*CWI* catheter wound infiltration group, *SAPB* serratus anterior plane block group, *NRS* visual analogic scale, *NIV* noninvasive ventilation, *ICU* intensive care unit

### Other results

**Fig. 2:**
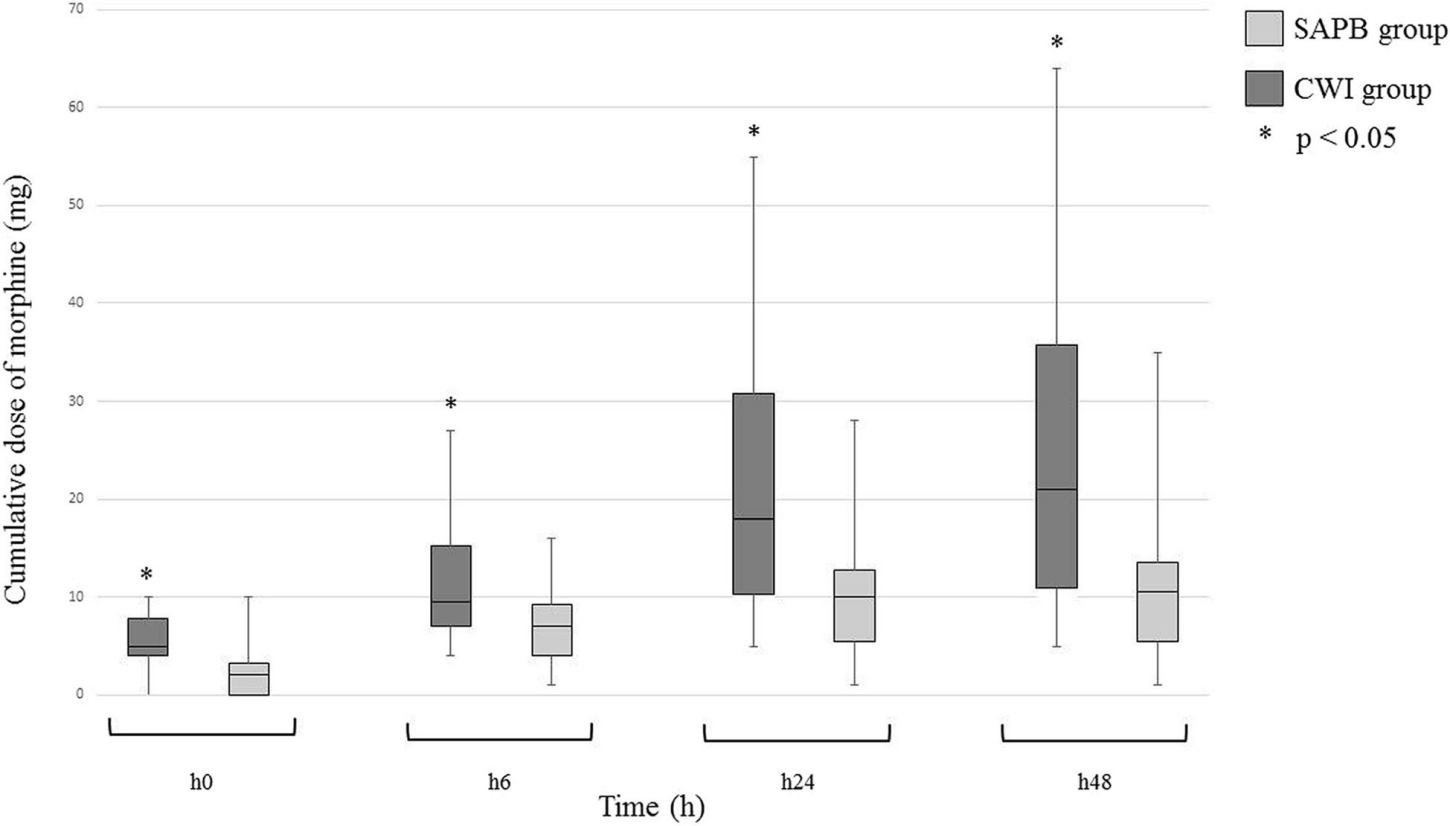
Morphine consumption during study period

Morphine titration (mg at h0) was significantly lower in the SABP group (5 [4–8] vs. 2 [0–4]; *p* < 0.01). The cumulative doses of morphine (mg) were significantly lower in the SAPB group at h6 (10 [7–15] vs 7 [4–10]; *p* = 0.01), h24 (18 [10–31] vs 10 [6–13]; p < 0.01) (Fig. 2). The pain evaluated by NRS before morphine titration was significantly lower in the SABP group (5 [4–6] vs. 0 [0–4]; p < 0.01). During the first 6 h and from the 6th to the 24th hour the pain tended to be lower in the SABP group but the difference was not statistically significant (Table 2). The lengths of stay (in hours) in ICU (41 [25–69] vs 24 [20–41]; *p* = 0.03) and in the hospital (8 [7–9] vs. 6 [5–8]; *p* = 0.03) are significantly shorter in the SAPB group. There was no statistically significant difference in the incidence of nausea and vomiting outcomes between the groups (*p* = 0.7). The need of NIV was reduce in SAPB group, 15% versus 23% in CWI group, but the result was not significate (*p* = 0.7).

## Discussion

Our study demonstrated that patients treated with SAPB had a significantly lower total morphine consumption during the first 48 h following minimally invasive heart surgery and lower NRS.

Analysis of morphine consumption in the different periods revealed that the difference was mainly related to a significantly lower dose of morphine titrated in SABP patients. In cumulative dose analysis, this difference persisted throughout the study period. This result was correlated with the pain experienced by the patient, because prior to morphine titration, NRS was significantly higher in the CWI group, accounting for the greater morphine consumption. Single-injection SAPB seems effective for early pain, but its effect is too short to provide adequate analgesia for 48 h after surgery. This result is in agreement with the study by Hetta et al., who reported a median duration of analgesia of six hours, and with the results of Blanco et al., who reported duration between 6 and 10 h [11, 17]. Median extubation time in our patients was 5 h; the morphine titration was therefore given towards the end of the SAPB efficacy period, thus explaining the lower doses injected at that time and the absence of any significant difference at subsequent time points.

CWI in patients who underwent thoracotomy was also investigated in the literature, but does not lead to a decrease in morphine consumption compared to a PCA alone [18]. The metameric spread of SAPB was not evaluated in our study; usually SAPB covers dermatomes T2 to T6, with variable degrees of cephalad spread reported in cadaveric studies [19, 20]. Nonetheless, the the surgical incision in MIHS, is systematically covered in the metameric spread of the block. Effectiveness of SAPB was also shown in a randomized-controlled trial for patients who undergo breast surgery. The analgesic result achieved with SAPB was good, compared with para-vertebral block [17]. The efficacy of SAPB has also been evaluated in thoracotomy and thoracoscopic surgery, with a reduction in pain and opioid consumption [21, 22].

Because our cohort was too small, we were unable to find a significant difference in nausea and vomiting and use of NIV. Okmen et al. did not demonstrate a difference for these criteria, but the number of patients was too small [22]. We can assume that painless patients had earlier post-operative rehabilitation with respiratory physiotherapy sessions in the first hours following extubation to reduce not only the respiratory morbidity associated with this type of intervention, but also the length of stay in the ICU and in hospital and thus the overall cost of care.

SAPB analgesia may have better hemodynamic stability compared to epidural analgesia, with no significant difference for NRS on the 24 h after surgery [23]. We did not observe any adverse events related to SAPB. This peripheral block seems easiest to apply and it is fast learning compared to perimedullary techniques. An experienced operator can perform the technique without assistance and without prolonging the overall duration of the anesthesia or the time between arrival in the ICU and extubation.

To overcome the short-lasting efficacy of SAPB, some authors have described the possibility of inserting a catheter into the SAPB space to allow continuous infusion of a local anesthetic for a predetermined time, after an initial bolus [14, 24, 25]. Such catheter insertion into the SAPB space for continuous ropivacaïne infusion could improve pain control during the first 48 h without opioid.

This retrospective study has several limitations. In particular, it’s retrospective study, without randomization and without blindness. However, the small size is quite close to the numbers of prospective randomized studies on SAPB. In the absence of preliminary data, we designed a retrospective comparative (exploratory) study with a convenience sample of 48 consecutive patients.

## Conclusion

SAPB may be effective in decreasing morphine consumption and pain after MIHS. A large double blind randomized trial is essential to confirm our results. Catheter insertion in the SAPB space with continuous infusion of a local anesthetic could be an interesting alternative for providing prolonged analgesia in the first days after surgery.

## Additional file

Additional file 1: Data of SAPB versus CWI study. Additional file contains demographic data of patients, morphine consumption and post operative pain in the SABP and CWI groups. (XLSX 26 kb)

## Abbreviations

AVR: Aortic valve replacement
BMI: Body mass index
CABG: Coronary Artery Bypass Graft Surgery
CNIL: Commission national de l’informatique et des libertées
CWI: Continuous wound infiltration
ICU: Intensive care unit
IQR: Interquartile range
MIHS: Minimally-invasive heart surgery
MVR: Mitral valve replacement;
NIV: Non invasive ventilation
NMT: Neuromuscular transmission
NRS: Numeric rating scale
PCA: Patient controlled analgesia
SAPB: Serratus anterior plane block
